# An evolutionary explanation of female‐biased sexual size dimorphism in North Sea plaice, *Pleuronectes platessa* L.

**DOI:** 10.1002/ece3.8070

**Published:** 2023-01-30

**Authors:** Fabian M. Mollet, Katja Enberg, David S. Boukal, Adriaan D. Rijnsdorp, Ulf Dieckmann

**Affiliations:** ^1^ Evolution and Ecology Program and Advancing Systems Analysis Program International Institute for Applied Systems Analysis (IIASA) Laxenburg Austria; ^2^ Wageningen Marine Research IJmuiden The Netherlands; ^3^ Department of Biological Sciences University of Bergen Bergen Norway; ^4^ Institute of Marine Research Bergen Norway; ^5^ Aquaculture and Fisheries Group Wageningen University Wageningen The Netherlands; ^6^ Complexity Science and Evolution Unit Okinawa Institute of Science and Technology Graduate University (OIST) Onna Japan; ^7^ Department of Evolutionary Studies of Biosystems The Graduate University for Advanced Studies (Sokendai) Hayama Japan; ^8^ Present address: Blueyou Consulting Ltd. Zürich Switzerland; ^9^ Present address: Department of Biological Sciences University of Bergen Bergen Norway; ^10^ Present address: Department of Ecosystem Biology, Faculty of Science University of South Bohemia Ceske Budejovice Czech Republic

**Keywords:** differential mortality model, energy allocation, female‐biased sexual size dimorphism, growth, life‐history evolution, maturation, reproductive investment, scramble competition

## Abstract

Sexual size dimorphism (SSD) is caused by differences in selection pressures and life‐history trade‐offs faced by males and females. Proximate causes of SSD may involve sex‐specific mortality, energy acquisition, and energy expenditure for maintenance, reproductive tissues, and reproductive behavior. Using a quantitative, individual‐based, eco‐genetic model parameterized for North Sea plaice, we explore the importance of these mechanisms for female‐biased SSD, under which males are smaller and reach sexual maturity earlier than females (common among fish, but also arising in arthropods and mammals). We consider two mechanisms potentially serving as ultimate causes: (a) Male investments in male reproductive behavior might evolve to detract energy resources that would otherwise be available for somatic growth, and (b) diminishing returns on male reproductive investments might evolve to reduce energy acquisition. In general, both of these can bring about smaller male body sizes. We report the following findings. First, higher investments in male reproductive behavior alone cannot explain the North Sea plaice SSD. This is because such higher reproductive investments require increased energy acquisition, which would cause a delay in maturation, leading to male‐biased SSD contrary to observations. When accounting for the observed differential (lower) male mortality, maturation is postponed even further, leading to even larger males. Second, diminishing returns on male reproductive investments alone can qualitatively account for the North Sea plaice SSD, even though the quantitative match is imperfect. Third, both mechanisms can be reconciled with, and thus provide a mechanistic basis for, the previously advanced Ghiselin–Reiss hypothesis, according to which smaller males will evolve if their reproductive success is dominated by scramble competition for fertilizing females, as males would consequently invest more in reproduction than growth, potentially implying lower survival rates, and thus relaxing male–male competition. Fourth, a good quantitative fit with the North Sea plaice SSD is achieved by combining both mechanisms while accounting for sex‐specific costs males incur during their spawning season. Fifth, evolution caused by fishing is likely to have modified the North Sea plaice SSD.

## Graphical abstract

The paper presents a model to evolutionarily explain the sexual size dimorphism with an eco‐genetic model adapted to detailed empirical data of North Sea plaice. Behavioral investments and diminishing fitness returns are considered as alternative explanations with its implication in individual energy allocation, and put into context with the ecology of the species.

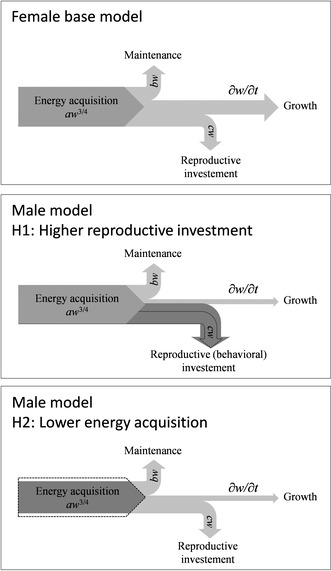

## 1 INTRODUCTION

### 1.1 Sexual size dimorphisms

Sexual size dimorphism (SSD) occurs when either males or females reach a larger adult body size than the other sex (Fairbairn et al., 2007). Male‐biased SSD (i.e., males being larger than females) is commonly observed in endotherms and in mammals in particular (Fairbairn et al., 2007). It has been extensively studied and is easily explained by adaptation also for ectotherms: Larger males have an advantage in male–male competition for females (for fishes, see, e.g., Emlen & Oring, 1977; Parker, 1992; Fleming & Gross, 1994). In contrast, the adaptive significance of female‐biased SSD (i.e., females being larger than males)—observed in various species of bony fish (e.g., Henderson et al., 2003; Pietsch, 1975; Rennie et al., 2008) but also in mammals (e.g., Fokidis et al., 2007), insects (e.g., Esperk et al., 2007), and spiders (e.g., Foellmer & Fairbairn, 2005)—is still poorly understood. SSD, in general, may be related to divergent gamete‐size evolution of males and females (anisogamy) and the resulting sex‐specific energy investments per gamete (Lehtonen & Kokko, 2010; Lessells et al., 2009; Parker, 1982): The larger eggs impose different energetic requirements than the smaller male gametes, which are minimized in size but maximized in number so as to compete for fertilizations (Bulmer & Parker, 2002; Lehtonen & Kokko, 2010; Parker, 1982). The evolutionary causes of anisogamy, however, remain unresolved (Klug et al., 2010; Kokko & Jennions, 2008). Also, SSDs exist in both directions (female‐biased and male‐biased), so even if the evolutionary causes of anisogamy were sufficiently understood, this could not directly help to understand SSDs. Instead, to explain the evolutionary causes of SSDs, the divergent selection pressures on, and lifestyles of, males and females have to be taken into account.

### 1.2 Proposed causes of female‐biased SSD

To explain female‐biased SSD, the Ghiselin–Reiss hypothesis (Ghiselin, 1974; Reiss, 1989) suggests that evolution will favor small males if male reproductive success is dominated by scramble competition among males for fertilization opportunities because smaller males require less energy and can thus devote more time to reproduction (finding females) than to growth (finding food). While males consequently acquire less food than females, their reproductive investments, including behavioral and physiological costs, might be similarly high as those of females, due to their higher cost of reproductive behavior and despite their lower cost of gamete production, thus causing males to be smaller than females. As a specification of the increased behavioral cost, the differential mortality model (DMM) has been proposed (Vollrath & Parker, 1992): Since males are the sex searching for fertilization opportunities, male adult mortality is higher, which relaxes male–male competition for females and thereby establishes a further evolutionary advantage for early‐maturing smaller males. Some studies have found empirical evidence for both hypotheses; for example, Blanckenhorn et al. (1995) reported that smaller body size in males correlated with indicators of higher success in scramble competition, and De Mas and Ribera (2009) found that smaller body size in males correlated with higher mortality. Other studies have not found such correlations (see, e.g., Foellmer & Moya‐Laraño, 2007). At any rate, an integrative understanding of the underlying mechanisms has remained elusive. For example, assuming that female body size is driven by fertility selection, Parker (1992) showed that weak sperm competition alone readily generates female‐biased SSD and that sex‐specific mortality (as considered by the DMM) further modifies the SSD (De Mas & Ribera, 2009). However, since Parker's model assumed that both sexes use the same patterns of energy acquisition and allocation, SSD in his model could arise only as a result of a sex‐specific age and size at maturation. In this study, we thus strive to establish an integrative understanding of the evolutionary basis of SSD using energy allocation as our conceptual starting point.

### 1.3 Expectations of SSD based on energy allocation

**FIGURE 1:**
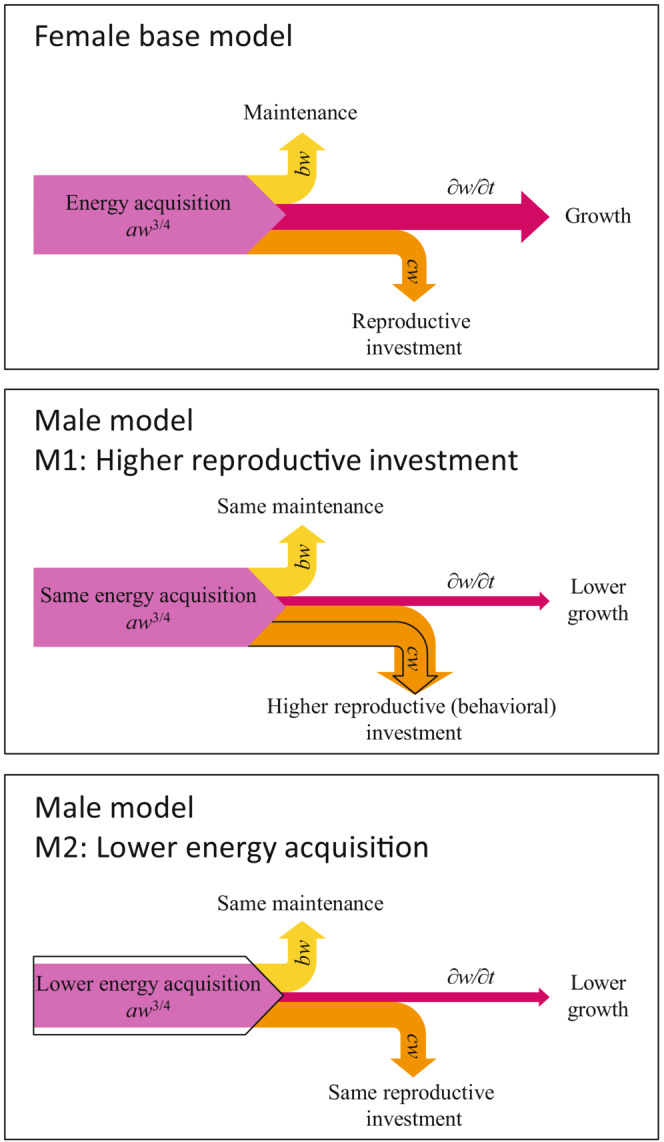
Energy‐allocation model used to explore potential mechanisms leading to female‐biased sexual size dimorphism (SSD). Acquired energy is first used for maintenance and reproductive investment, with the remainder being available for somatic growth. The resultant female base energy flows are shown in purple, yellow, red, and orange (top panel). Male growth might be reduced if (M_1_) more energy is spent for reproductive investment than in females due to an additional male behavioral investment (middle panel), or (M_2_) less energy is acquired than in females while the other metabolic rates stay equal (bottom panel). The resultant different male energy allocations are shown in orange and purple, respectively, superimposed on the female base energy allocations shown by black outlines. Complementing the mechanism M_1_, time costs (M_1a_) and mortality costs (M_1b_) of prolonging the male spawning season are also considered

Energy allocation describes how individuals channel their acquired energy toward growth, maintenance, and reproduction (von Bertalanffy & Pirozynski, 1952; West et al., 2001). Sex‐specific differences in growth and length at age may result from differential maturation and from differences in energy acquisition or reproductive investment (Figure 1). In general, female‐biased SSD can thus result either from males investing more energy in reproductive behavior (Figure 1, middle panel) or from males acquiring less energy (Figure 1, bottom panel). Lower energy acquisition, or growth efficiency, in males has indeed been observed in fish species with female‐biased SSD, and a higher male reproductive investment has been suggested as a potential explanation of female‐biased SSD (Henderson et al., 2003; Rennie et al., 2008). Although the connection was not made explicit in those earlier studies, the mechanisms of lower male energy acquisition and of higher male reproductive investment are both compatible with the Ghiselin–Reiss hypothesis, which predicts that males would stay smaller, thus acquiring less energy, and invest more in reproduction than growth, if their reproductive success was dominated by scramble competition for fertilizing females. Here, we build on these mechanisms by examining the evolution of SSD under general sex‐specific energy allocations and maturation patterns. In short, we study the following two, not mutually exclusive, general mechanisms for explaining female‐biased SSD (Figure 1):

- Males invest more in reproductive behavior. This may happen if they need to compete for females and the energy required for the associated behavior is then no longer available for somatic growth. Such behavioral cost may be complemented by a time cost and/or a mortality cost when male reproductive behavior implies less time being available for feeding and/or a higher exposure to predators, respectively.
- Males acquire less energy. This may happen if reproductive success in males is less dependent on body size and overall reproductive investment than in females. In this case, males will forage less and hence avoid predation and/or disease‐related mortality. If mating opportunities are limited in space and time, male reproductive success may become largely uncoupled from male body size, which can be approximated by considering diminishing returns on male reproductive investment with increasing body size.

### 1.4 Implications of SSD for fisheries

Sexual size dimorphism may have important implications for the sustainable harvesting of fish populations. Differences in growth rates imply differences in the productivity of male and female stock components (Beverton & Holt, 1957). Since fishing typically is size‐selective, it is likely also to be sex‐specific, which affects a stock's long‐term sustainable yield and has implications for the proper assessment of a stock’s status and its fishery (Kell & Bromley, 2004). In addition, fisheries selection may elicit evolutionary responses (e.g., Dieckmann et al., 2009; Heino et al., 2013, 2015; Heino & Dieckmann, 2009; Jørgensen et al., 2007; Laugen et al., 2014), which SSD may thus render sex‐specific. It is therefore important to understand the evolutionary mechanisms that can lead to female‐biased SSD. Among fishes, the flatfish North Sea plaice *Pleuronectes platessa* L. is a prominent example of female‐biased SSD (Rijnsdorp & Ibelings, 1989; Van Walraven et al., 2010). Due to its commercial importance, North Sea plaice ranks among the best‐studied fish species, with exceptional quantitative data being available on its life history, and thus for understanding the observed SSD.

### 1.5 Scope of this study

As no integrative assessment of the relative merits of the aforementioned potential mechanistic causes of the life‐history evolution of female‐biased SSD has as yet been offered in the literature, we evaluate their importance in North Sea plaice using an eco‐genetic modeling approach (Dunlop, Heino, et al., 2009). Our model quantitatively describes the ecology and inheritance of growth, maturation, and reproduction, as well as the life‐history trade‐offs between growth and mortality and between reproduction and mortality. The same model has previously been used without sex structure and SSD to explore the consequences of fisheries‐induced evolution and to study management opportunities for mitigating the impacts of fisheries‐induced evolution on sustainable harvest (Mollet, Dieckmann, et al., 2016; Mollet, Poos, et al., 2016). Here, we expand this approach by allowing for sex structure and SSD, to explore which mechanisms are required to model the male life history consistently with the available life‐history data in general and with the observed female‐biased SSD in particular. We also assess the possible effects of fishing on the North Sea plaice SSD. After presenting our findings for North Sea plaice, we discuss how these can be extended to other species and to male‐biased SSD.

## 2 MATERIALS AND METHODS

### 2.1 Modeling approach

Addressing the research question of understanding the selection pressures that lead to different energy‐allocation patterns in males and females resulting in SSD (Figure 1) requires examining how the energy‐allocation traits determining body size—describing energy acquisition, maturation schedule, and reproductive investment—evolve over time. For accomplishing this objective, three major challenges have to be met simultaneously by the chosen modelling approach.

First, the literature on the Ghiselin–Reiss hypothesis and on the differential mortality model demonstrates that an integrative understanding of the evolutionary causes of SSD cannot be established when the involved traits, trade‐offs, life‐history processes, and environmental determinants are addressed either only qualitatively or only partially. In particular, ecological and demographic dynamics have to be modeled sufficiently faithfully to predict resultant selection pressures reliably. It is therefore important to use a modeling approach that allows studying the underlying life‐history complexity both quantitatively and comprehensively.

Second, the evolution of the involved traits is determined by the corresponding fitness landscape, which in turn depends on environmental conditions and a population's trait composition. The latter dependence means that the evolution of a trait has feedback on its own evolution and on the evolution of all other traits, which is technically known as frequency‐dependent selection. It is therefore important to use a modeling approach that accounts for eco‐evolutionary feedback.

Third, it is important to use a modelling approach that can be calibrated to the empirical data available for a real population. To account for life‐history complexity, eco‐evolutionary feedback, and calibration requirements, we use an individual‐based eco‐genetic model. In comparison with other possible approaches, this offers several advantages: (a) An individual‐based model is understandable at the individual level, where the modeled trade‐offs indeed apply; (b) realistic ecological dynamics with sufficient life‐history complexity can be specified to determine selection pressures; (c) eco‐evolutionary feedback is readily included; (d) model parameters and outputs can be calibrated to real populations; and (e) in higher‐dimensional spaces spanned by a population's continuous states and traits, individual‐based models can be more computationally efficient than models using partial differential or integro‐differential equations implemented through a grid of compartments (as the vast majority of the latter tend to be empty; Dunlop, Heino, et al., 2009).

### 2.2 North Sea plaice data

Plaice is sexually dimorphic: Females mature at larger body sizes and older ages than males and subsequently also grow to larger adult body sizes (Rijnsdorp & Ibelings, 1989). During spawning in winter, male and female North Sea plaice cease feeding, but males remain twice as long in spawning condition than females (Rijnsdorp & Ibelings, 1989). The instantaneous mortality rate for males during spawning is about twice as high as for females, suggesting a difference in behavior that increases the exposure of males to predators and to fishing gear (Beverton, 1964; Rijnsdorp, 1993). Since North Sea plaice has been exploited intensively for more than a century (Rijnsdorp & Millner, 1996), its current life‐history characteristics are likely affected by fisheries‐induced evolution (Heino et al., 2015; Jørgensen et al., 2007): In particular, the age at maturation has decreased, and reproductive investment has increased (Grift et al., 2003, 2007; Van Walraven et al., 2010).

Our life‐history model, specified below, is simultaneously fitted to three independent life‐history datasets focusing, respectively, on growth, maturation, and reproduction: (a) length at age, described by age‐specific somatic weights, (b) maturation length at age, described by the age‐specific midpoints of a probabilistic maturation reaction norm (PMRN), and (c) reproductive investment at age, based on gonad weights and migration costs and described by an age‐specific somatic‐weight equivalent. Details and data sources are presented in Appendix 2.

### 2.3 Model description

We use an individual‐based eco‐genetic model (Dunlop, Heino, et al., 2009; see also Dunlop, Baskett, et al., 2009; Eikeset et al., 2013, 2016, 2017; Enberg et al., 2009, 2010; Marty et al., 2014; Mollet, Dieckmann, et al., 2016; Mollet, Poos, et al., 2016; Okamoto et al., 2009; Thériault et al., 2008; Wang & Höök, 2009) calibrated to North Sea plaice. Below we provide an overview of the key model features; more details are presented in Appendix 1. All model variables are listed in Table A1 and all model parameters in Table A2.

**FIGURE 2:**
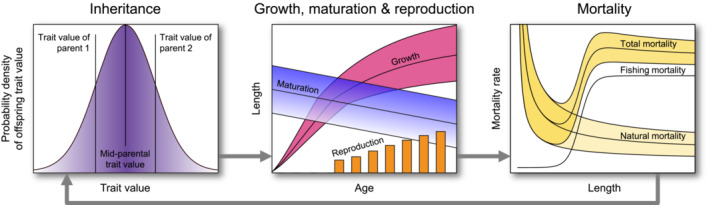
Illustration of the individual‐based eco‐genetic model underlying this study's analyses. Male and female individuals inherit and express evolving traits based on which they undergo life cycles involving growth, maturation, mate selection, reproduction, and mortality. Inheritance is determined by the principles of quantitative genetics. Growth and reproduction are determined by the energy‐allocation model (see Equation (1.1), (1.2) and Appendix 1: Equations A1–A2). Maturation is determined by a probabilistic maturation reaction norm (PMRN) describing the probability of maturation as a function of age and length (see Appendix 1: Equations B1–B3). Mortality comprises natural mortality and fishing mortality, while natural mortality comprises effects from several causes (see Appendix 1: Equations A8–A14). The resultant population is updated annually and feeds back on individual life histories through frequency dependence and density dependence. Most of the life‐history processes are modeled stochastically

Our model follows cohorts of superindividuals (Scheffer et al., 1995) throughout their lifetime and determines their survival probability and reproductive success in annual time increments, based on their genetic trait values and their phenotypic expression of these. The model accounts for sex structure and sex‐specific trait expression: Male and female individuals inherit a male‐specific and a female‐specific value of each trait, with only the value corresponding to their sex being phenotypically expressed. The modeled evolving traits expressed in males or females determine an individual's energy‐acquisition rate a, reproductive‐investment rate c, and probabilistic maturation reaction norm (PMRN) intercept u (Table 1, Figure 2). The evolving traits expressed in males further include an individual's spawning duration $t_{\text{sg}}$, determining the residence time annually spent on the spawning ground; for females, $t_{\text{sg}}$ is fixed at 1/8 year.

**TABLE 1:** Average phenotypic values of the four evolving life‐history traits in the unexploited population (${\text{F}}_{\text{max}}=0.00\;{\text{year}}^{‐1}$) and the exploited population (${\text{F}}_{\text{max}}=0.37\;{\text{year}}^{‐1}$) at the respective evolutionary equilibria for the various considered mechanisms explaining sexual size dimorphism (SSD). Evolving traits: spawning duration $t_{\text{sg}}$, energy‐acquisition rate *a*, reproductive‐investment rate *c*, and PMRN intercept *u*. The third column explains how the considered mechanisms, or combinations thereof, are impacting the modeling of male life histories

			Unexploited population				Exploited population			
Sex	Mechanism	Modeling implications
$t_{\text{sg}}$ [year]	a [g^1/4^ year^−1^]	c [year^−1^]	u [cm]	$t_{\text{sg}}$ [year]	a [g^1/4^ year^−1^]	c [year^−1^]	u [cm]
Female	Absence of all mechanisms M_1_, M_1a_, M_1b_, and M_2_.	None.	0.125	5.59	0.233	50.6	0.125	5.88	0.421	25.4
Male	M_1_: male fitness affected by diminishing returns of behavioral (time) investments.	Spawning duration $t_{\text{sg}}$ evolves. Male reproductive success $\nu_{m}$ influenced by diminishing returns $h(t_{\text{sg}})$ of spawning duration, while $f(\gamma_{i})=1$ (Equation (3.1), (3.2), (3.3), (3.4)).	0.282	5.77	0.288	50.8	0.342	5.94	0.454	28.8
Male	M_1a_: male fitness affected by growth costs of behavioral (time) investments.	Spawning duration $t_{\text{sg}}$ evolves. Male reproductive success $\nu_{m}$ influenced by diminishing returns $h(t_{\text{sg}})$ of spawning duration, while $f(\gamma_{i})=1$ (Equation (3.1), (3.2), (3.3), (3.4)). Male growth reduced according to spawning duration $t_{\text{sg}}$ (see Appendix 1: Equation A.1.2).	0.242	5.78	0.267	48.6	0.272	5.94	0.372	29.8
Male	M_1b_: male fitness affected by mortality costs of behavioral (time) investments.	Spawning duration $t_{\text{sg}}$ evolves. Male reproductive success $\nu_{m}$ influenced by diminishing returns $h(t_{\text{sg}})$ of spawning duration, while $f(\gamma_{i})=1$ (Equation (3.1), (3.2), (3.3), (3.4)). Male mortality raised according to spawning duration (Equation (4.1), (4.2))	0.228	5.88	0.239	59.4	0.314	6.00	0.411	33.4
Male	M_1ab_: male fitness affected by growth and mortality costs of behavioral (time) investments.	Same as above while male growth reduced according to spawning duration $t_{\text{sg}}$ (see Appendix 1: Equation A.1.2) and male mortality raised according to spawning duration (Equation (4.1), (4.2)).	0.192	5.891	0.213	58.7	0.242	6.01	0.331	33.2
Male	M_2_: male fitness affected by diminishing returns of reproductive (energy) investments.	Male reproductive success $\nu_{m}$ influenced by diminishing returns $f(\gamma_{i})$ of reproductive investment, while $h(t_{\text{sg}})=1$ (Equation (3.1), (3.2), (3.3), (3.4)).	0.125	5.50	0.232	33.6	0.125	2.85	0.471	8.12
Male	M_1ab2_: combination of all mechanisms M_1_, M_1a_, M_1b_, and M_2_.	Spawning duration $t_{\text{sg}}$ evolves. Male reproductive success $\nu_{m}$ influenced by diminishing returns $h(t_{\text{sg}})$ of spawning duration and by diminishing returns $f(\gamma_{i})$ of reproductive investment (Equation (3.1), (3.2), (3.3), (3.4)). Male growth reduced according to spawning duration $t_{\text{sg}}$ (see Appendix 1: Equation A.1.2) and male mortality raised according to spawning duration (Equation (4.1), (4.2)).	0.175	5.28	0.230	34.3	0.223	5.21	0.32	15.6

The energy available for somatic growth at a given body weight *w* is given by the difference between the energy‐acquisition rate $aw^{\alpha }$ and the energy expenditure rate bw for maintenance and, after becoming mature, the energy expenditure rate cw for reproduction (Von Bertalanffy & Pirozynski, 1952; West et al., 2001). We consider the evolution of the rates of energy acquisition (a) and of reproductive investment (c), respectively, while the maintenance rate (b) is assumed to be constant. Based on metabolic theory (West et al., 1997) and species‐specific estimates (Fonds et al., 1992), we assume that the rate of energy acquisition scales with somatic weight w to the power of α=3/4, whereas the rates of maintenance and reproductive investment scale with w to the power of 1. This leads to the following dynamics for the body weight of individuals,

(1.1) $$\frac{dw}{dt}=aw^{3/4}‐bw\;\text{for juveniles,}$$

(1.2) $$\frac{dw}{dt}=aw^{3/4}‐\left(b+c\right)w\;\text{for}\;\text{adults},$$

where t denotes time. The energy‐acquisition rate *a* depends on population density, implying density‐dependent growth (see Appendix 1: Equation 4). Equation (1.1), (1.2) determines the discrete‐time dynamics required for a model with annual time steps, describing the somatic weight $w_{t+1\text{year}}$ as a function of the somatic weight $w_{t}$ (Appendix 1: Equation A1).

Maturation is determined by a probabilistic maturation reaction norm (PMRN; see Appendix 1: Equation A5) describing the dependence of the probability of maturing on an individual's age and length (Dieckmann & Heino, 2007; Heino & Dieckmann, 2008; Heino et al., 2002; Stearns & Koella, 1986). The age‐specific PMRN midpoints $l_{\text{p}50,t}$, that is, the body lengths at which the probability of maturing equals 50% at age t, are assumed to follow a linear function of age with an intercept u and a slope s,

(2) $$l_{\text{p}50,t}=u+st.$$

The PMRN intercept u is allowed to evolve, while the PMRN slope s is assumed to be fixed (see Appendix 1: Equations B1–B3 for the empirical estimation of the midpoint function). Body lengths are related to body weights according to a fixed allometric relationship (see Appendix 1: Equation A3).

After maturation, individuals reproduce in annual mating events, during which a focal individual's probability to produce offspring depends on its reproductive success relative to all other individuals (see Appendix 1: Equations A6 and A7). The reproductive investment γ, itself a function of the energy‐allocation model (see Appendix 1: Equation A2), is described by a somatic‐weight equivalent and can be interpreted to comprise all investments that contribute to annual reproduction, including gamete production and behavioral investments such as spawning migration and spawning behavior. Reproductive success is a sex‐specific function of this investment, and the difference in these functions between the sexes is crucial for explaining SSD (see below).

Natural mortality is applied during each year and includes predation mortality, which decreases with body size and increases with foraging behavior as described by the energy‐acquisition rate a (growth‐survival trade‐off), reproduction mortality, which depends on the relative energy loss due to reproduction (reproduction‐survival trade‐off), and starvation mortality, which occurs when the maintenance costs bw cannot be covered by an individual (see Appendix 1: Equations A8 and A10–A13). Since the observed life history of North Sea plaice is the result of adaptation to fishing, our model also includes the sex‐specific size‐dependent patterns of fishing mortality characteristic for North Sea plaice (see Appendix 1: Equations A9 and A14).

Midparental genetic values determine inherited genetic traits (see Appendix 1: Equation A15), and environmental variability is adjusted so as to match the heritability levels of life‐history traits expected in fish (Roff, 1991). Processes of maturation, mate selection, trait inheritance, trait expression, and mortality are all modeled stochastically. For continuous outcomes (e.g., phenotypic trait values), this is done by randomly drawing the realized value from a normal distribution with a mean describing the expected value and a standard deviation describing the expected environmental variation (see Appendix 1: Equation A16). For binary outcomes (e.g., maturation or death), it is done by randomly drawing a value between 0 and 1 from a uniform distribution and realizing the binary outcome if this value is smaller than the outcome's probability.

### 2.4 SSD mechanisms

We evaluate the capacity of the two not mutually exclusive mechanisms described in the Introduction to explain female‐biased SSD (Figure 1, Table 1). Based on the empirical evidence that all of these mechanisms are likely to apply, the model parameters were fitted (see below) using mechanisms M_1ab2_ (see below), before exploring their separate effects on female‐biased SSD by separately adding the respective mechanism to the female base model (Table 1).

The divergent male life history corresponding to each considered mechanism or combination thereof is assumed to evolve from sex‐specific differences in reproductive success in conjunction with sex‐specific differences in natural mortality and fishing mortality. While the relative reproductive success of a female individual is directly proportional to its reproductive investment γ (see Appendix 1: Equation A7), the relative reproductive success $\nu_{m,i}$ of a male individual i is given by

(3.1) $$\nu_{m,i}=\frac{f\left(\gamma_{i}\right)h\left(t_{\text{sg},i}\right)}{\sum_{j=1}^{N_{m}}f\left(\gamma_{j}\right)h\left(t_{\text{sg},j}\right)},$$

where f(γ) and $h(t_{\text{sg}})$ are functions capturing the diminishing returns of the reproductive investment γ and the spawning duration $t_{\text{sg}}$, respectively, on reproductive success:

(3.2) $$f\left(\gamma \right)={\left(1+\gamma_{50}/\gamma \right)}^{‐1},$$

(3.3) $$h\left(t_{\text{sg}}\right)={\left(1+t_{\text{sg},50}/t_{\text{sg}}\right)}^{‐1}.$$

These functions describe a decreasing marginal gain in reproductive success from reproductive investments in terms of γ and $t_{\text{sg}}$ in males, respectively, whereas reproductive success in females linearly increases with reproductive investment (see Appendix 1: Equation A7 and Figure 6). Assuming that the rate of reproductive activity of males is constant during the male spawning duration $t_{\text{sg}}$, we consider their reproductive‐investment rate $c_{m}$ to be proportional to $t_{\text{sg}}$,

(3.4) $$c_{m}=\theta t_{\text{sg}}.$$

For both sexes, the natural mortality rate M is given by a baseline mortality rate $m_{b}$, a predation mortality rate $m_{p}$ caused by foraging (growth‐survival trade‐off), a mortality rate $m_{r}$ due to reproduction (reproduction‐survival trade‐off), and a starvation mortality rate $m_{s}$ (see Appendix 1: Equations A10–A13). For both sexes, the fishing mortality rate F applies (see Appendix 1: Equation A14). Because of their spawning activity, males suffer from an additional predation mortality rate ${\text{ς}}_{\text{sg}}w^{\eta }$ and from an additional fishing‐mortality rate $F(\tau_{\text{sg}}‐1)$ during the male spawning duration $t_{\text{sg}}$,

(4.1) $$M_{m}=m_{b}+m_{p}+m_{r}+m_{s}+\varsigma_{\text{sg}}w^{\eta }(t_{\text{sg}}/1\text{year}),$$

(4.2) $$F_{m}=F\left(1+\left(\tau_{\text{sg}}‐1\right)(t_{\text{sg}}/1\text{year})\right).$$

Given these differences in male life history, the following mechanisms are tested for their potential to explain female‐biased SSD (Table 1):

- Mechanism M_1_: Diminishing returns on reproductive success of prolonged male spawning. We consider a diminishing return of prolonged male spawning duration $t_{\text{sg}}$, that is, a decreasing marginal gain as male spawning duration is increased (Equation 3.3).
- Mechanism M_1a_: Time costs of prolonged male spawning. In addition to mechanism M_1_, we consider that male behavioral reproductive activity comes at a time cost. As feeding ceases during spawning (Rijnsdorp & Ibelings, 1989), prolonging $t_{\text{sg}}$ in males reduces their time available for growth (see Appendix 1: Equation A1.2).
- Mechanism M_1b_: Mortality costs of prolonged male spawning. In addition to mechanism M_1_, we consider that male behavioral reproductive activity comes at a mortality cost. Due to elevated exposure to predators and to fishing gear during spawning, higher mortality rates apply to males on the spawning ground; prolonging $t_{\text{sg}}$ in males thus reduces their survival (Equation (4.1), (4.2)).
- Mechanism M_2_: Diminishing returns of raised male reproductive investment. We consider a diminishing return of raised male reproductive investment, that is, a decreasing marginal gain as male reproductive investment is increased (Equation 3.2).

M_1ab_ refers to the combination of mechanisms M_1_, M_1a_, and M_1b_, while M_1ab2_ refers to all four mechanisms being applied together.

### 2.5 Model calibration

The parameterization of our model was carried out in three steps. In a first step, parameters for which independent estimates are available were either taken from the literature or directly estimated from empirical data on age, size, and maturity from Dutch market samples and scientific surveys (Table A2).

In a second step, the remaining parameters were fitted for the female life history (Table A2), separately for the historic period (around 1900) and the present period (around 2000), so as to minimize the mean Δ of squared relative deviations for ages t=1,…,10 years of model‐predicted (subscript M) from empirically observed (subscript E) population‐averaged age‐specific body weights ${\bar{w}}_{t}$, age‐specific PMRN midpoints ${\bar{l}}_{\text{p50},t}$, and age‐specific relative reproductive investments ${\bar{r}}_{t}={\bar{\gamma_{t}/w}}_{t}$,

(5) $$\Delta =\frac{1}{10}\sum_{t=1}^{10}{\left(\frac{{\bar{w}}_{t,M}‐{\bar{w}}_{t,E}}{{\bar{w}}_{t,E}}\right)}^{2}+\frac{1}{6}\sum_{t=1}^{6}{\left(\frac{{\bar{l}}_{\text{p50},t,M}‐{\bar{l}}_{\text{p50},t,E}}{{\bar{l}}_{\text{p50},t,E}}\right)}^{2}+\frac{1}{5}\sum_{t=6}^{10}{\left(\frac{{\bar{r}}_{t,M}‐{\bar{r}}_{t,E}}{{\bar{r}}_{t,E}}\right)}^{2}.$$

The age ranges 1–10 years, 1–6 years, and 6–10 years represent the growth phase, maturation phase, and reproduction phase, respectively. Each of the three sums of squares is divided by the number of terms in the sum so as to give the calibrations of growth, maturation, and reproduction equal relative importance. As empirical observations on reproductive investments are unavailable for the historic period, the third term above was omitted when calculating Δ for that period. Model‐predicted values were obtained after ensuring that the population was at evolutionary equilibrium. Parameter combinations minimizing Δ were determined using a grid‐based search (Table A2). The average natural mortality rate at age 6 year was set to $M=0.1\;{\text{year}}^{‐1}$ in accordance with the ICES stock assessment (ICES, 2011). The density dependence of energy‐acquisition rates (see Appendix 1: Equation A4) was assumed to be absent (negligible) in the heavily exploited (and thus, low‐density) present population state. For both the historic period (subscript H) and the present period (subscript P), the maximum fishing mortality rate ${\text{F}}_{\text{max}}$ was included in the estimated parameters (yielding ${\text{F}}_{\text{max},H}=0.27\;{\text{year}}^{‐1}$ and ${\text{F}}_{\text{max},P}=0.37\;{\text{year}}^{‐1}$, respectively).

In the first two calibration steps, the female traits were calibrated by modeling the males as females. In the third step, the male traits were introduced and calibrated to distinguish male from female life history. The male‐specific life‐history parameters ($t_{\text{sg},50}$, $\gamma_{50}$, and θ) were fitted using mechanisms M_1ab2_ based on the female‐specific parameter settings in the present population obtained from the second step, by minimizing Δ (Equation 5) for males using a grid‐based search (Table A2).

## 3 RESULTS

### 3.1 Effects of all mechanisms considered together

**FIGURE 3:**
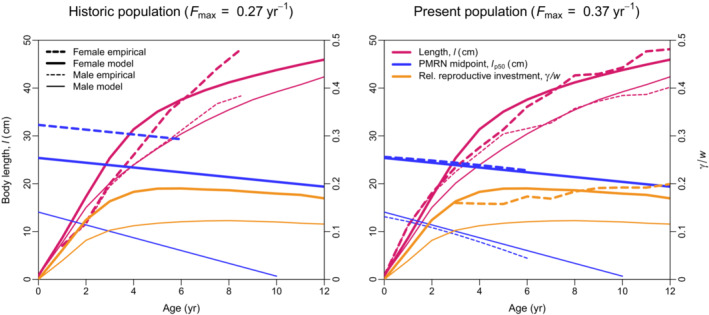
Comparison of model predictions with empirical observations. Average lengths at age (red; left vertical axes), PMRN midpoints at age (blue; left vertical axes), and relative reproductive investments at age (orange; right vertical axis) from empirical data (dashed lines) and model predictions using mechanisms M_1ab2_ (continuous lines) for females (thick lines) and males (thin lines) of the historic population (left panel) and the present population (right panel). To be comparable with gonadosomatic indices, reproductive investments were scaled to the energy‐equivalent female gonadic weights

The empirically observed and model‐predicted life‐history characteristics for mechanisms M_1ab2_ are displayed in Figure 3 for the historic population and the present population. These results show that our model is capable of eco‐evolutionarily reproducing the empirically observed life histories of males and females, not only for the present population but also for the historic population. Our model thus is the first to recover the SSD in North Sea plaice based on a process‐based eco‐evolutionary life‐history model maximally informed by empirical data.

### 3.2 Effects of sex‐specific behavioral reproductive investments and diminishing returns

**FIGURE 4:**
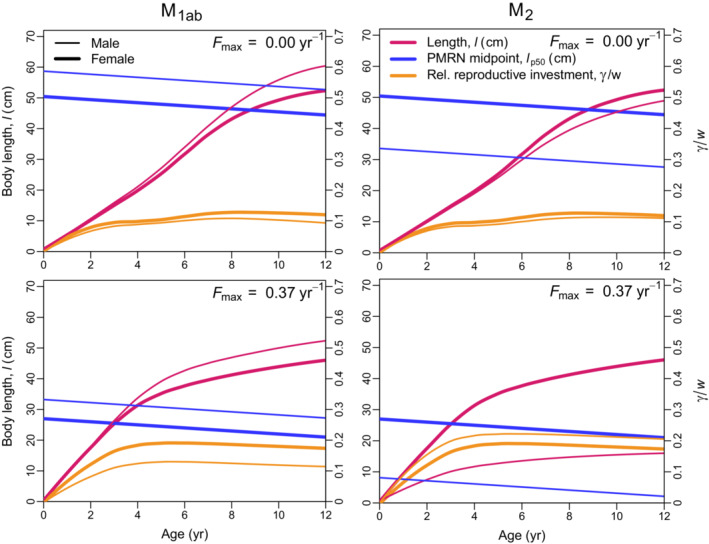
Lengths at age (red), PMRN midpoints (blue), and relative reproductive investments γ/w as proportions of somatic weight (orange) of females (thick lines) and males (thin lines) resulting from the hypothesized mechanisms potentially leading to female‐biased SSD: Benefits of increased male behavioral investments are traded off against time costs and mortality costs (M_1ab_, left panels) or the diminishing return of male reproductive investment (M_2_, right panels). The effects of these mechanisms are shown for the unexploited population (${\text{F}}_{\text{max}}=0.00\;{\text{year}}^{‐1}$, upper panels) and the exploited population (${\text{F}}_{\text{max}}=0.37\;{\text{year}}^{‐1}$, lower panels)

**FIGURE 5:**
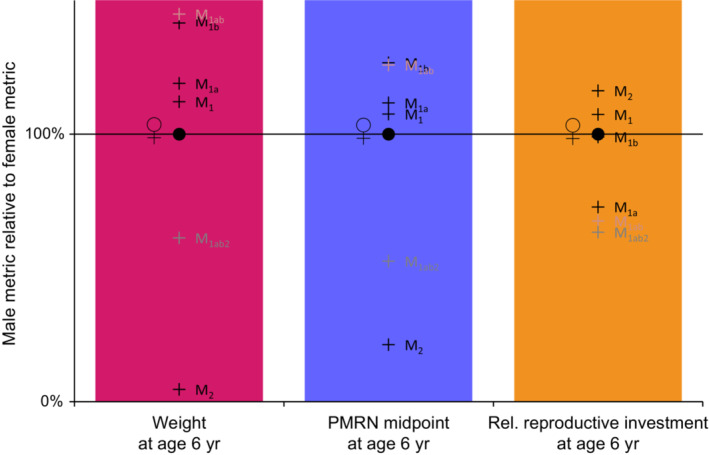
Effects of the six mechanisms or mechanism combinations M_1_, M_1a_, M_1b_, M_1ab_, M_2_, and M_1ab2_ (see text) on three metrics of SSD. The three panels show (from left to right) the weight at age 6 year ($w_{\mathrm{6year}}$), the PMRN midpoint at age 6 year ($l_{\text{p}50,6\text{year}}$), and the relative reproductive investment at age 6 year ($\gamma_{\mathrm{6year}}/w_{\mathrm{6year}}$). The effects of each considered mechanism or mechanism combination on the male metrics (crosses) are shown relative to the corresponding female metrics (filled circles at 100%)

The results for the effects of the single mechanisms considered in isolation reveal that the SSD in North Sea plaice can be recovered and understood by mechanism M_2_ alone, but not by mechanism M_1_ alone (Figures 4 and 5, Table 1). Adding the mechanisms M_1a_ and M_1b_, which account for the time costs (M_1a_) and mortality costs (M_1b_) of behavioral reproductive investments (more time spent on the spawning ground), we observe that the energy‐acquisition rate in males increases to compensate for the higher energy demand, leading to larger males, a male‐biased SSD, and a slight upward shift in the male PMRN (Figures 4 and 5, Table 1). These effects are not in agreement with the empirical observations.

The diminishing returns of male reproductive investment, as described by mechanism M_2_, result in decreased energy‐acquisition rates, earlier maturation, and, consequently, a significantly smaller length at age and lower PMRN in males (Figures 4 and 5, Table 1). This finding applies to the exploited population as well as to the unexploited population (Figure 4, Table 1). Under exploitation ($F_{\text{max,P}}=0.37\;{\text{year}}^{‐1}$), the fitness advantages of the smaller size and earlier maturation in males are amplified for the commonly observed ages.

We thus suggest that the female‐biased SSD in North Sea plaice can best be understood as a consequence of reduced energy acquisition and earlier maturation caused by diminishing returns of reproductive investment in males, and not of a higher demand for relative reproductive investment in males, as had previously been proposed (e.g., Henderson et al., 2003; Rennie et al., 2008).

### 3.3 Effects of sex‐specific time costs and mortality costs of reproductive investments

When the increase in male reproductive behavioral investment incurs time costs due to a reduced growing season (mechanism M_1a_) and mortality costs due to reduced feeding activities (mechanism M_1b_), this results in increased energy acquisition and delayed maturation in males (Tables 1 and 2, Figures 4 and 5). The mortality costs (mechanism M_1b_) have the stronger effect on increased size and delayed maturation; these effects are further increased by exploitation, since the mortality costs increase with exploitation. In contrast, the time costs (mechanism M_1a_) have a stronger effect on reducing the relative reproductive investment γ/w, while their effect on maturation is ambiguous, depending on the level of exploitation (not shown). As a consequence, these two effects amplify the effects toward male‐biased SSD relative to mechanism M_1_ alone. Due to their positive effect on male size, mechanisms M_1a_ and M_1b_ improve the fit to the empirical data when combined with mechanism M_2_, as they are responsible for a lower male relative reproductive investment γ/w under exploitation ($F_{\text{max,P}}=0.37\;{\text{year}}^{‐1}$).

**TABLE 2:** Population averages of emergent metrics describing SSD in the unexploited, historic, and present populations considering mechanisms M_1ab2_. In parentheses, the SSD in each metric is summarized by the ratio of the male value to the female value, $x_{m}/x_{f}$

Metric	Symbol [unit]	Sex	Unexploited population		Historic population		Present population	
Asymptotic length	$l_{\infty }$ [cm]	f	59.8		49.8		48.7	
		m	56.9	(95%)	45.9	(92%)	47.9	(98%)
Length at age 6 year	$l_{\mathrm{6year}}$ [cm]	f	30.4		37.2		37.6	
		m	27.1	(89%)	29.5	(79%)	30.4	(81%)
Age at maturation	$t_{\text{mat}}$ [year]	f	9.6		5.6		4.1	
		m	7.1	(74%)	4.4	(79%)	3.1	(76%)
Length at maturation	$l_{\text{mat}}$ [cm]	f	48.1		35.2		31.2	
		m	32.8	(68%)	24.2	(69%)	19.9	(64%)
Relative reproductive investment at age 6 year	$\gamma_{\mathrm{6year}}/w_{\mathrm{6year}}$ [‐]	f	0.105		0.167		0.182	
		m	0.097	(92%)	0.112	(67%)	0.110	(60%)

### 3.4 Effects of sex‐specific exploitation

Comparing the effects on the evolving traits among the unexploited population ($F_{\text{max,P}}=0.00\;{\text{year}}^{‐1}$), the historic population ($F_{\text{max,P}}=0.27\;{\text{year}}^{‐1}$), and the present population ($F_{\text{max,P}}=0.37\;{\text{year}}^{‐1}$) allows us to formulate expectations for the effects of fishing. With increased exploitation rates, realized energy‐acquisition rates *a* increase (because density‐dependent competition is relaxed; the effects on the genetic energy‐acquisition rates may differ), reproductive‐investment rates *c* increase (and, thus, spawning duration $t_{\text{sg}}$ in males increases), and the PMRN shifts to lower levels in both sexes (Table 1). The pace of the life histories increases due to increased exploitation rates, leading to an increase in size at younger ages (e.g., at age 6 year), but to a decrease in size at older ages (e.g., at age 10 year; Figure 3).

Because the amplitudes of the life‐history responses to increased fishing mortality differ between the sexes, our results show that fishing affects the amplitude of the sexual dimorphism in adult size (SSD), maturation, and reproductive investment, although the change may not be monotonic with respect to fishing mortality, so that the direction of change may vary during a history of increased exploitation (Table 2). First, SSD, expressed in terms of the ratio of the asymptotic body size ($l_{\infty }$) between male and females, increases in magnitude due to fishing in the historic population relative to the unexploited population and subsequently decreases in the present population. Second, sexual dimorphism in the onset of maturation decreases in the historic population relative to the unexploited population and subsequently increases in the present population. Third, reproductive investment shows a consistent increase in sexual dimorphism in response to higher exploitation rates.

## 4 DISCUSSION

### 4.1 SSD through lower energy acquisition or higher reproductive investment?

Our model predicts female‐biased SSD only when there are diminishing returns of male reproductive investment (mechanism M_2_). It can thus be concluded that smaller male size arises as a consequence of lower energy‐acquisition rates in males. The alternative mechanism of a higher reproductive investment through additional behavioral costs or spawning behavior in males (mechanisms M_1_, M_1a_, and M_1b_) leads to higher energy‐acquisition rates in males, delayed maturation in males, and a male‐biased SSD. The extent to which higher behavioral reproductive investments are compensated for by higher energy‐acquisition rates will be influenced by the strength of the growth‐survival trade‐off. Predation mortality accelerates with the energy‐acquisition rate (see Appendix 1: Equation A11), but we have found that increasing this acceleration does not affect the main findings (not shown). We therefore expect that higher reproductive investments will generally be supported by higher energy‐acquisition rates. On the other hand, we expect that lower energy‐acquisition rates are a general consequence if fitness returns from increased reproductive investment are diminishing and high energy‐acquisition rates are costly. Under size‐selective fishing, this cost is obviously amplified.

### 4.2 Causes and implications of diminishing fitness returns

The evolutionary force leading to reduced energy acquisition in males, and thus to female‐biased SSD, results from the diminishing returns of reproductive investment. Several mechanisms may lead to such diminishing returns. First, particularly in seasonal environments, the time window in which fish can reproduce successfully is restricted (Cushing, 1990), resulting in a temporal limitation of mating opportunities. In contrast to females, males could vary their spawning duration, but the marginal gains for a male to increase its spawning duration and its reproductive investment decrease, due to the limitation of mating opportunities. These diminishing returns are thus one possible interpretation of the Ghiselin–Reiss hypothesis according to which small males evolve if male reproductive success is a function of scramble competition for mating opportunities with females (Ghiselin, 1974; Reiss, 1989). The rate at which returns on reproductive investments are diminishing depend on the intensity of male–male competition or sperm competition: For example, if sperm competition is weak, a focal male needs relatively less sperm to fertilize the same amount of eggs, resulting in more strongly diminishing returns of investing in sperm production.

### 4.3 Scramble competition and the differential mortality model

Scramble competition for limited mating opportunities has been argued to increase male–male competition and therefore lead to increased male body size (e.g., Parker, 1992). Male–male competition can be inferred from the operational sex ratio (OSR), that is, the local ratio of sexually active males to fertilizable females (Kvarnemo & Ahnesjo, 1996). Higher OSRs (more active males per fertilizable female) will increase scramble competition and select for males with higher male reproductive investment. Higher male reproductive investment, however, typically comes at a mortality cost and might therefore compensate for the skewed OSR (Kokko & Jennions, 2008). The competition‐induced mortality corresponds to the differential mortality model (DMM; Vollrath & Parker, 1992), which we account for in our model through additional male mortality as function of increased reproductive activity (i.e., prolonged spawning duration $t_{\text{sg}}$). The additional male mortality skews OSRs toward females and therefore relaxes gamete competition and sexual selection (Okuda, 2011; Parker, 1992; Vollrath & Parker, 1992), from which smaller males might be expected—which is the basis of the expectation resulting from the DMM. The scramble competition caused by a male‐biased OSR might thus typically be neutralized because the OSR induces frequency‐dependent selection for increased male investment, and since this investment increases male mortality, the OSR is pushed back toward 1 again (Kokko & Jennions, 2008) and does therefore not necessarily lead to an expectation of larger males. Yet, the mortality effect results in larger males due to competition‐independent effects. The additional male mortality leads to increased energy acquisition and delayed maturation: Males take the risk of increased mortality on the spawning ground (resulting from both natural mortality and fishing mortality) only if this risk is balanced by fitness gains through high reproductive success attained at relatively larger sizes. In this sense, the DMM has to be rejected as the direct cause of female‐biased SSD. Yet, the DMM might be part of the justification of the diminishing returns: Since additional male mortality skews OSRs and consequently relaxes gamete competition, it might be one of the mechanisms due to which male reproductive returns are diminishing. It is, however, not the mortality effect itself that leads to smaller males but its potential to neutralize scramble competition. Male–male competition might also be offset if males develop alternative mating tactics such as sneaking (Parker, 1990), but such behaviors were not in the scope of this study.

In the case presented here, mortality causes males to evolve to larger sizes because it selectively applies only to adults. The result would not be the same if the mortality applied equally over the entire lifespan. The mortality effect found here is in line with the general finding that increasing adult mortality results in delayed maturation (Ernande et al., 2004; Law & Grey, 1989) and with similar results from eco‐genetic models in which later maturation is observed when a fishery mainly harvests on a stock's spawning ground compared to harvesting the same population on its feeding ground (Dunlop, Baskett, et al., 2009; Heino et al., 2015; Jørgensen et al., 2009).

### 4.4 Extrapolation of results

**FIGURE 6:**
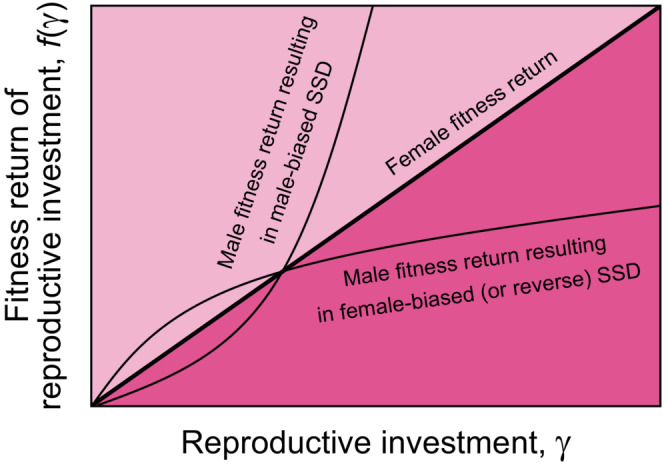
Differences in fitness returns of reproductive investment between males and females and consequences for SSD. The sex with the higher eventual steepness of the fitness return at increasing reproductive investments will evolve to larger body size. For the sake of illustration, the female fitness return is shown to increase linearly with reproductive investment

Diminishing returns of male reproductive investment (mechanism M_2_) might be the evolutionary cause of female‐biased SSD in other species as well, in which mating opportunities are limited and/or limited male–male competition occurs (see Webb & Freckleton, 2007, for a review of female‐biased SSD). In contrast, mating systems with strong male–male competition for access to females ready to reproduce may be conceived as systems in which males receive an increasing return from reproductive investment; consequently, a male‐biased SSD will evolve. The evolutionary cause of SSD might generally lie in the difference in reproductive investment returns in the broad sense between males and females (Figure 6): The sex with the eventually steeper fitness returns will evolve to have larger body size. Factors in female life history might also determine the difference in steepness between males and females (Figure 6): Returns on female reproductive investment will, for instance, increase with body size, as their fecundity and offspring survival increase with body size (e.g., Trippel & Neil, 2004); multiple egg batches spawned over the spawning period will increase the probability that at least some larvae will encounter favorable environmental conditions; and high gamete survival will enhance the effect of low sperm competition and thus accentuate the diminishing returns (Lehtonen & Kokko, 2010). In summary, all of the following factors will contribute to diminishing fitness returns in males relative to females, and hence, result in female‐biased SSD: Limitation of mating opportunities, low level of sperm competition (possibly mediated partly through differential mortality), high fertilization probability, and maternal size effects.

Furthermore, it might be a general pattern that mortality rates between the sexes differ due to reproduction. Differences in sex‐specific mortality rates will likely be due to reproductive behavior, and the different sexes will likely behave equally in their juvenile phases (e.g., Henderson et al., 2003). Mortality in the adult part of a population has been shown to delay maturation and increase body sizes evolutionarily, both in theory and in practice (Ernande et al., 2004; Heino et al., 2015; Jørgensen et al., 2007; Law & Grey, 1989). That differential male mortality—although partly justifying a diminishing return in reproductive investment by relaxing competition—leads to larger size and delayed maturation, might therefore also be a general result.

### 4.5 Model fit

The above findings, in conjunction with the empirical evidence for the life history of North Sea plaice, corroborate the understanding that the combination M_1ab2_ of mechanisms is the best choice for modeling and understanding the SSD in this population. Mechanism M_2_ is required to obtain a female‐biased SSD, and mechanisms M_1a_ and M_1b_ are needed to match the observed reproductive investments γ, which would otherwise be too high.

Under exploitation, the lower relative reproductive investment γ/w in males is caused by the time costs (mechanism M_1a_) and mortality costs (mechanism M_1b_) of spawning but not by the diminishing‐return mechanism M_2_ (Figures 4 and 5). Reproductive investment γ depends on metabolic rates (a, c) and the onset of maturation ($t_{\text{mat}}$, Equation 5), and since the energy‐acquisition rate *a* increases under mechanisms M_1a_ and M_1b_, the decrease in relative reproductive investment γ/w must be due to a lower reproductive‐investment rate c, driven by the costs imposed by mechanisms M_1a_ and M_1b_. Although not causing SSD, the mechanisms of time costs (M_1a_) and mortality costs (M_1b_) therefore help to improve the fit with the empirical data under mechanisms M_1ab2_.

For sizes around maturation, the model fit for males could be improved further by including a switch in energy acquisition after maturation. While males might grow fast before maturation to outgrow the predation‐size window and thus reduce their size‐dependent predation risk, they may reduce their energy‐acquisition rate thereafter (Henderson et al., 2003; Rennie et al., 2008). Modeling such a switch in energy acquisition after maturation would therefore make biological sense and improve our model's fit to the empirical data by avoiding the overestimation of male size around maturation, but it would also add complexity to our already complex model, to an extent that was deemed unnecessary for the scope of this paper.

### 4.6 Effect of fishing

Our results are consistent with predictions from similar models that fisheries‐induced evolution leads to a faster pace of life, namely to faster growth, higher reproductive investment, and earlier onset of maturation (e.g., Dunlop, Heino, et al., 2009; Enberg et al., 2009). Exploitation affects SSD by differentially affecting the evolution of these traits in males and females, due to the sex‐specific trade‐offs shaping SSD. Such differential evolution will, however, depend on the balance between the mortalities caused by spawning, predation, and exploitation, and might further differ because of other species‐specific causes. The effects of the energy‐acquisition rate a and the reproductive‐investment rate c on body size are opposite to each other, and changes in SSD thus depend on the strength of selection on each.

If SSD was simply defined based on the ratio of the asymptotic size between the sexes, one would have to conclude that SSD always decreases due to exploitation (Table 2). However, in contrast to the hypothetical asymptotic size, which is never reached, such a monotonic dependence on fishing mortality might not apply at intermediate ages, to which a substantial fraction of individuals might survive (e.g., age 6 year; Table 2). Also, the sexual dimorphism in length at maturation does not increase monotonically when fishing mortality is raised (Table 2). Such nonmonotonic effects might be due to threshold effects on the fitness landscape and the correlation of traits. For example, as soon as maturation evolves to occur at sizes below the size at which fish are vulnerable to fishing, an additional selection pressure is expected to kick in for not growing beyond this size, which can be achieved by adaptations of energy acquisition and reproductive investment.

Our model calibration assumes that the historic and current populations of North Sea plaice are at evolutionary equilibrium under a constant fishing mortality and selectivity. However, the ongoing decrease in the PMRN of North Sea plaice (Grift et al., 2003, 2007; Van Walraven et al., 2010) suggests that the current population is still under fishing‐induced selection pressures, and thus keeps evolving. Relaxing the assumption of evolutionary equilibrium would affect the calibration and consequently also retrospective or prospective predictions. Yet, since we focused on the causes of SSD in the time window within which the model was calibrated, the assumption of evolutionary equilibrium is not expected to distort the results and conclusions for SSD.

## 5 CONCLUSIONS

Our results may provide an evolutionary explanation for female‐biased SSD in species that have a similar mating system as North Sea plaice. We reject the hypothesis that smaller males evolve due to higher activity costs during reproduction and suggest that female‐biased SSD is instead caused by diminishing returns of increased reproductive investments in males relative to females. The evaluated mechanisms provide an evolutionary explanation of the Ghiselin–Reiss hypothesis (Ghiselin, 1974; Reiss, 1989) and elucidate that the differential mortality model (DMM; Vollrath & Parker, 1992) is unlikely as a direct cause of female‐biased SSD. Our study presents the first eco‐genetic model fitted in such detail to empirical estimates of age‐specific empirical data of size, maturation probability, and reproductive investment for males and females. Since our model captures key demographic processes and can reproduce empirical data for both present and historic populations of North Sea plaice, it provides a method for assessing the evolutionary impacts caused by the North Sea plaice fishery (Heino et al., 2015; Jørgensen et al., 2007). The modeling framework introduced here could therefore become a powerful tool for exploring and evaluating alternative management measures to mitigate fisheries‐induced evolution, supporting a modern Darwinian approach to fisheries management (Laugen et al., 2014).

## CONFLICT OF INTEREST

None declared.

## AUTHOR CONTRIBUTIONS

**Fabian M. Mollet:** Conceptualization (supporting); data curation (equal); formal analysis (lead); investigation (supporting); methodology (supporting); resources (lead); software (lead); validation (lead); visualization (lead); writing—original draft (lead); writing—review and editing (lead). **Katja Enberg:** Conceptualization (supporting); methodology (equal); resources (supporting); software (supporting); supervision (supporting); validation (supporting); writing—original draft (supporting). **David S. Boukal:** Conceptualization (supporting); investigation (equal); methodology (supporting); supervision (supporting); writing—review and editing (supporting). **Adriaan D. Rijnsdorp:** Conceptualization (supporting); data curation (equal); funding acquisition (supporting); investigation (supporting); methodology (supporting); project administration (lead); supervision (lead); validation (supporting); writing—review and editing (supporting). **Ulf Dieckmann:** Conceptualization (lead); formal analysis (lead); funding acquisition (lead); investigation (supporting); methodology (lead); project administration (equal); supervision (equal); validation (equal); visualization (supporting); writing—review and editing (equal).

## Figures and Tables

**FIGURE A1:**
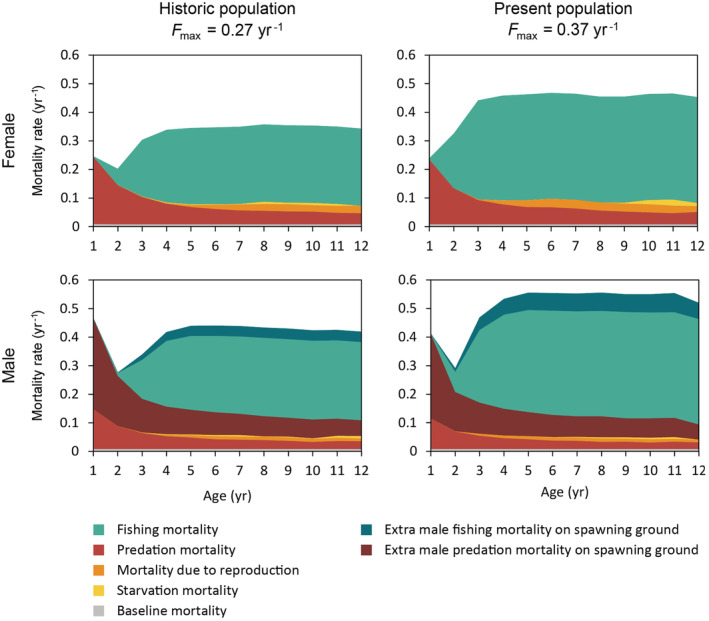
Composition of average mortality at age. Instantaneous mortality rates are shown for females (top row) and males (bottom row) in the historic population (left column) and present population (right column). Mortality components: (a) fishing mortality (Equation A14), (b) extra fishing mortality experienced by males due to their spawning activities (difference between Equations 4.2 and A9), (c) predation mortality (Equation A11), (d) extra predation mortality experienced by males due to their spawning activities (differences between Equations 4.1 and A8), (e) mortality due to reproduction (Equation A12), (f) starvation mortality (Equation A13), and (g) baseline mortality (Equation A10). Males suffer from elevated overall mortality rates, as the net result of five effects: their fishing mortality is elevated by their spawning activities (b), their predation mortality is elevated by their spawning activities (d), their predation mortality is elevated by their smaller lengths at age (c), their predation mortality is reduced by their lower energy‐acquisition rates (c), and their mortality due to reproduction is reduced by their lower reproductive investments (e)

**FIGURE A2:**
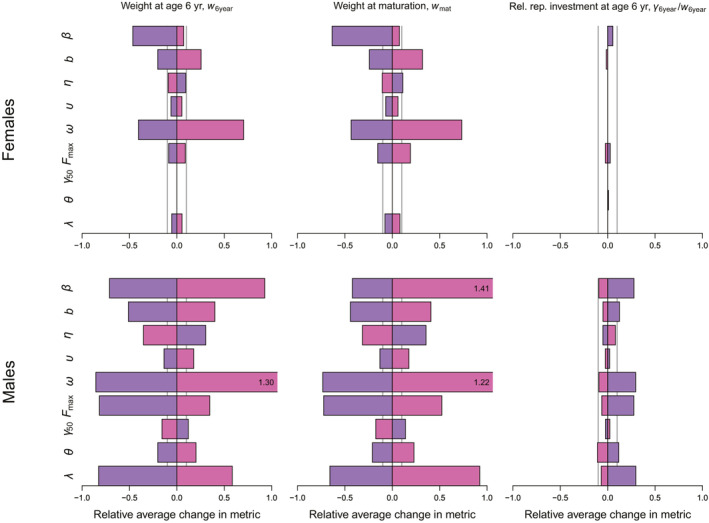
Sensitivity analysis of key model results. The three columns show the relative effects of changes in model parameters on three metrics: weight $w_{6\text{year}}$ at age 6 year (left column), weight $w_{\mathrm{mat}}$ at maturation (middle column), and relative reproductive investment $\gamma_{6\text{year}}/w_{6\text{year}}$ at age 6 year (right column) for females (top row) and males (bottom row). Light‐purple histogram bars: effects of a 10% decrease in the focal parameter. Dark‐purple histogram bars: effects of a 10% increase in the focal parameter. Only those parameters are displayed that have an effect of more than 10% (with these thresholds indicated by the gray vertical lines) for at least one of the shown metrics of the female or male life history

## Data Availability

Empirical data as well as code examples for model runs are available through Dryad: https://doi.org/10.5061/dryad.vx0k6djs8.

## APPENDIX 1

### Model details

#### Energy allocation

An individual's growth is derived from metabolic energy allocation (Von Bertalanffy & Pirozynski, 1952, West et al., 2001) assuming an allometric relation of energy acquisition $aw^{3/4}$ with body weight w that fits well theoretically (West et al., 1997) and empirically to North Sea plaice (Fonds et al., 1992) as described in the main text (Equation 1). Reproduction is prioritized over growth. If the acquired energy cannot cover maintenance costs, the individual neither grows nor reproduces and experiences starvation mortality (Equation A13).

To predict individual growth in each year, the time‐continuous energy‐allocation model (Equation 1) is used to obtain a time‐discrete model with annual time steps, by expressing the somatic weight $w_{t+1\text{year}}$ at age t+1year as a function of the somatic weight $w_{t}$ at age t at the start of the growing season (for a list of all model variables and parameters, see Tables A1 and A2, respectively),

(A1.1)
$$w_{t+1\text{year}}^{1/4}=\frac{a}{b}-\left(\frac{a}{b}-w_{t}^{1/4}\right)e^{-b\left(1\text{year}\right)/4}\;\text{for juveniles},$$

(A1.2)
$$w_{t+1\text{year}}^{1/4}=\frac{a}{b+c}-\left(\frac{a}{b+c}-w_{t}^{1/4}\right)e^{-\left(b+c\right)\left(1\text{year}-t_{\mathrm{sg}}\right)/4}\;\text{for adults}.$$

**TABLE A1:** Model variables, including evolving traits and emergent individual‐level or population‐level characteristics changing with the evolving traits and/or the environment.

	Symbol	Description	Unit
Structure	t	Age: time since birth	year
	w	Somatic weight	g
	l	Body length	cm
Evolving traits			
Energy allocation	$a_{g}$, a	Genetic (potential) and phenotypic (realized) size‐specific energy‐acquisition rates	g^1/4^ year^−1^
	$c_{g}$, c	Genetic and phenotypic size‐specific reproductive‐investment rates	year^−1^
	$u_{g}$, u	Genetic and phenotypic probabilistic maturation reaction norm (PMRN) intercepts	cm
	$t_{\mathrm{sg},g}$, $t_{\mathrm{sg}}$	Genetic and phenotypic spawning durations	year
Emergent traits			
Maturation	$p_{\mathrm{mat}}\left(t,l\right)$	Age‐ and size‐specific probability of maturation	–
	$l_{p50.t}$	Age‐specific PMRN midpoint: length l at which $p_{\mathrm{mat}}\left(t,l\right)$ at age t equals 50%	cm
	d	PMRN width: length increment between the maturation probabilities $p_{\mathrm{env}}$ and $1-p_{\mathrm{env}}$ defining the maturation envelope	cm
Reproduction	γ	Annual size‐specific reproductive investment in terms of its energy‐equivalent somatic weight: energy spent on reproduction, including gonadic as well as behavioral investments	g
	$\nu_{f,i}$	Female reproductive success of individual i	–
	$\nu_{m,i}$	Male reproductive success of individual i	–
	$f\left(\gamma \right)$	Fitness return from reproductive energy investment	–
	$h\left(t_{\mathrm{sg}}\right)$	Fitness return from reproductive time investment	–
Mortality	$M_{f}$, $M_{m}$	Instantaneous natural mortality rates in females and males	year^−1^
	$F_{f}$, $F_{m}$	Instantaneous fishing mortality rates in females and males	year^−1^
	$m_{b}$	Instantaneous baseline mortality rate	year^−1^
	$m_{p}$	Instantaneous predation mortality rate (growth‐survival tradeoff)	year^−1^
	$m_{r}$	Instantaneous mortality rate due to reproduction (reproduction‐survival tradeoff)	year^−1^
	$m_{s}$	Instantaneous starvation mortality rate	year^−1^
Inheritance	$x_{g},x$	Genetic and phenotypic values of trait x	Trait‐specific
	$x_{f},x_{m}$	Maternal and paternal values of trait x	Trait‐specific
	$\mu_{g,x}$	Population mean of genetic trait x	Trait‐specific
	$\sigma_{g,x}^{2}$	Population variance of genetic trait x	Trait‐specific
	$\sigma_{e,x}^{2}$	Part of phenotypic variance caused by environmental variability of trait x	Trait‐specific
Abundance	B	Biomass of individuals (<25 cm) competing for resources	g
	$N_{f}$	Number of adult females	–
	$N_{m}$	Number of adult males	–
	$N_{R}$	Number of recruits surviving to age t=1year	–
Fit	Δ	Deviation between model predictions and empirical estimates	–

**TABLE A2:** Model parameters. Reference: parameter value taken from the indicated reference. Direct estimation: parameter value estimated directly from individual‐level data (on size, age, and maturity) from market samples and scientific surveys (see Appendix 2). Calibration: no empirical data available, parameter value calibrated to provide the best fit to population‐level data (see Appendix 3).

	Symbol	Description	Source	Value	Unit
Energy allocation & Maturation	b	Size‐specific maintenance rate (Equations 1, A1 and A2)	Mollet et al. (2010)	0.6	year^−1^
	$s_{m}$ $s_{f}$	Male PMRN slope (Equation 2) Female PMRN slope (Equation 2)	Direct estimation	–0.5 –1.34	cm year^−1^
	$\epsilon /u_{g}$	Scaling of PMRN width d (Equation B3), relative to the genetic PMRN intercept $u_{g}$	Direct estimation	0.11	–
	k β	Parameters of length‐weight allometry (Equation A3)	Rijnsdorp (1990)	0.01 3.0	g cm^−3^ –
	$\delta_{1}$ $\delta_{2}$	Parameters of density dependence of growth (Equation A4)	Calibration^a^	9.6 10^−7^ 10.82	g^−1^ –
Reproduction	$w_{\mathrm{egg}}$	Egg weight (Equation A6)	Direct estimation	4.2 10^−3^	g
	$r_{1}$ $r_{2}$	Parameters of stock‐recruitment relationship (Equation A6)	ICES (2008)	8 10^3^ 1 10^6^	– –
	$\gamma_{50}$	Reproductive investment resulting in half‐maximal success of energy investment in reproduction (Equation 3.2)	Calibration^b^	47.0	g
	$t_{\mathrm{sg},50}$	Spawning period resulting in half‐maximal success of time investment in reproduction (Equation 3.3)	Calibration^b^	0.11	year
	θ	Proportionality constant linking spawning duration to reproductive‐investment rate (Equation 3.4)	Calibration^b^	1.45	year^−2^
Natural mortality	$M_{f}$	Instantaneous total natural mortality rate for an average‐sized female at age 6 year (Equation A8)	Beverton (1964) ICES (2008)	0.1	year^−1^
	$\varsigma_{\mathrm{sg}}$	Parameter to differentiate male and female instantaneous natural mortality rates on the spawning ground due to increased male activity (Equation 4.1)	Beverton (1964)	1.41	g^1/4^ year^−1^
	υ	Allometric coefficient of instantaneous baseline predation mortality rate (Equation A10)	Calibration^a^	1.25 10^−4^	g^1/4^ year^−1^
	η	Allometric exponent of instantaneous predation mortality rate (Equation A10)	Peterson and Wroblewski (1984) Brown et al. (2004)	–0.25	–
	$m_{0}$	Instantaneous size‐independent baseline mortality rate (Equation A10)	Calibration^a^	0.009	year^−1^
	ω	Parameter in growth‐survival tradeoff (Equation A11)	Calibration^a^	1.28	g^−1/4^ year
	χ	Parameter in reproduction‐survival tradeoff (Equation A12)	Calibration^a^	9.4	–
	ρ	Proportionality constant in starvation mortality rate (Equation A13)	Schultz et al. (1999)	5.0	–
Fishing mortality	$\tau_{\mathrm{sg}}$	Parameter to differentiate male and female instantaneous fishing mortality rates on the spawning ground due to increased male activity (Equation 4.2)	Rijnsdorp (1993)	1.75	–
	$F_{\max,P}$ $F_{\max,H}$	Maximum instantaneous fishing mortality rate in the present population (P) and the historic population (H) assuming evolutionary equilibrium (Equation A14)	Calibration^a^ Calibration^a^	0.37 0.27	year^−1^ year^−1^
	ϕ	Fishing‐selectivity constant (Equation A14)	Van Beek et al. (1983)	0.594	cm^−1^
	λ	Mesh‐size selection factor (Equation A14)	Van Beek et al. (1983)	2.2	–
	ψ	Mesh size (Equation A14)	Van Beek et al. (1983)	8.0	cm
Inheritance	CV	Coefficient of variation of evolving traits (Equations A15 and A16)	Grift et al. (2003) Mollet et al. (2010)	0.1	–
	$h^{2}$	Heritability of evolving traits (Equation A16)	Roff (1991)	0.24	–

^a^ These parameters were calibrated based on the listed directly estimated parameter values by applying the following search grid: $F_{\max}$ = 0, 0.1, …, 0.5 year^−1^; ω = 1, 1.01, …, 1.5 g^−1/4^ year; and χ = 5, 5.2, …, 15. For each parameter combination on this grid, υ and $m_{0}$ were determined assuming that the natural mortality rate $M_{f}$ equals 0.1 year^−1^ (ICES 2011) and that the mortality rate $m_{r}$ due to reproduction and the predation mortality rate $m_{p}$ equally contribute to $M_{f}$ for a female with average traits at age 6 year. To estimate $\delta_{1}$ and $\delta_{2}$, a search grid was applied for the reduction of energy acquisition due to density dependence in the historic population state: *a*/*a*_g_ = 0.5, 0.51, …, 1. Assuming that there was no such reduction in the present population state, $\delta_{1}$ and $\delta_{2}$ were then determined from the two corresponding instances of Equation A4.

^b^ These male‐specific parameters were calibrated based on all other listed parameter values by applying the following search grid: $t_{\mathrm{sg},50}$ = 0, 0.1, …, 0.4 year; $\gamma_{50}$ = 0, 1, …, 100 g; and θ = 1.00, 1.01, …, 2.00 year^−2^.

No energy is allocated to somatic growth in adults during the spawning duration $t_{\mathrm{sg}}$, and maintenance during the spawning duration is covered by stored energy reserves.

An individual's reproductive investment $\gamma_{t+1\text{year}}$ at age t+1year consists of its gonadic and behavioral investment and is obtained by integrating its reproductive‐investment rate, which is proportional to its weight, over the growing season according to Equation 1,

(A2)
γt+1year=∫tt+1yearcwt′dt′=cb+cwt−wt+1year+4a3b+cwt3/4−wt+1year3/4+2a2b+c2wt1/2−wt+1year1/2+4a3b+c3wt1/4−wt+1year1/4+4a4b+c4lna−b+cwt1/4a−b+cwt+1year1/4.

For males, c is replaced with $c_{m}$ in Equations A1 and A2, to reflect their extra behavioral cost of reproduction.

The following allometric length‐weight relationship applies to postspawning individuals whose gonads and energy reserves have been emptied,

(A3)
$$w_{t}=kl_{t}^{\beta }.$$

Since the model is implemented in annual time steps, variation in the length–weight relationship within the year is irrelevant.

Growth is density‐dependent because of intraspecific competition for food. The energy‐acquisition rate a can maximally reach its genetically determined potential value $a_{g}$ and decreases with the modeled population's total biomass B,

(A4)
$$a=\frac{a_{g}}{1+{\left(\delta_{1}B\right)}^{\delta_{2}}}.$$

#### Maturation

An individual's probabilistic maturation reaction norm (PMRN) is determined by an intercept u and a slope s defining the age‐specific PMRN midpoints $l_{p50,t}$ according to Equation 2. The probability of maturing at a given age t is assumed to increase logistically with length $l_{t}$ around $l_{p50,t}$,

(A5)
$$p_{\mathrm{mat}}\left(t,l,\left(t\right)\right)=\frac{1}{1+e^{-\left(l_{t}-l_{p50,t}\right)/\epsilon }}.$$

For simplicity, we assume that the modeled population's mean slope s and ratio $\epsilon /u_{g}$ remain constant as the genetic PMRN intercept $u_{g}$ is evolving.

#### Reproduction

The number of recruits $N_{R}$ surviving to age t=1year is given by a Beverton–Holt–type stock–recruitment relationship and depends on total fecundity, given by the sum of the reproductive investments $\gamma_{i}$ of all $N_{f}$ mature females $i=1,\ldots ,N_{f}$, assuming a constant egg weight $w_{\mathrm{egg}}$,

(A6)
$$N_{R}=\frac{r_{1}}{1+r_{2}w_{\mathrm{egg}}/\sum_{i=1}^{N_{f}}\gamma_{i}}.$$

Reproductive success increases linearly with reproductive investment $\gamma_{i}$ in females,

(A7)
$$\nu_{f,i}=\frac{\gamma_{i}}{\sum_{j=1}^{N_{f}}\gamma_{j}},$$

but nonlinearly in males, for which additionally the spawning duration *t*_sg_ matters (Equation 3): since the probability of successful mating decreases before and after peak spawning and since mating opportunities are limited, there are diminishing returns of increasing the spawning duration *t*_sg_ (Equation 3.3) and of increasing the reproductive energy investment γ (Equation 3.2) in males.

#### Mortality

Fish are exposed to both natural mortality and fishing mortality; see Figure A1 for the resultant sex‐specific mortality patterns.

The natural mortality rate $M_{f}$ of females is given by a baseline mortality rate $m_{b}$ caused by diseases and predation (Equation A10), a predation mortality rate $m_{p}$ caused by foraging (growth‐survival tradeoff; Equation A11), a mortality rate $m_{r}$ due to reproduction (reproduction‐survival tradeoff; Equation A11), and a starvation mortality rate $m_{s}$ (Equation A13), while the fishing mortality rate $F_{f}$ of females is given by a mortality rate F accounting for size‐selective fishing (Equation A14),

(A8)
$$M_{f}=m_{b}+m_{p}+m_{r}+m_{s},$$

(A9)
$$F_{f}=F.$$

Data and theory suggest that the predation mortality rate in marine systems allometrically scales with body weight (Peterson & Wroblewski, 1984; Brown et al., 2004; Savage et al., 2004). The baseline mortality rate is therefore assumed to be composed of a size‐independent component due to diseases, including parasites, and an allometric size‐dependent component due to predation,

(A10)
$$m_{b}=m_{0}.$$

Since higher potential energy‐acquisition rates $a_{g}$ induce higher foraging rates and thus a higher risk of exposure to predators, the predation mortality rate increases with $a_{g}$,

(A11)
$$m_{p}=\upsilon w^{\eta }e^{\omega a_{g}},$$

Depletion of stored energy due to reproduction may result in a lower survival probability (e.g., Hutchings, 1994). The mortality rate $m_{r}$ due to reproduction is therefore assumed to increase with the stored relative reproductive energy investment γ/w,

(A12)
$$m_{r}=m_{0}\left(e^{\chi \gamma /w}-1\right).$$

If individuals do not acquire sufficient energy to cover their maintenance costs, i.e., if $aw^{3/4}-\mathrm{bw}\leq 0$, they starve at an instantaneous mortality rate proportional to the rate of energy loss per unit of somatic weight,

(A13)
$$m_{s}=\max\left(0,,,-,\rho \frac{aw^{3/4}-\mathrm{bw}}{w}\right).$$

The fishing mortality rate F at body length *l* is determined by a maximum $F_{\max}$ and mesh selection with mesh size ψ, selection factor λ, and selectivity constant ϕ (Sparre & Venema, 1998),

(A14)
$$F=\frac{F_{\max}}{1+e^{-\phi \left(l-\lambda \psi \right)}}.$$

Due to their spawning activity, males are more vulnerable and, during their spawning duration $t_{\mathrm{sg}}$, suffer from additional predation mortality (their baseline predation mortality rate is increased by a factor $1+\left(\varsigma_{\mathrm{sg}}/\upsilon \right)\left(t_{\mathrm{sg}}/1\text{year}\right)$; Equation 4.1) and additional fishing mortality (their fishing mortality rate is increased by a factor $1+\left(\tau_{\mathrm{sg}}-1\right)\left(t_{\mathrm{sg}}/1\text{year}\right)$; Equation 4.2).

#### Inheritance and expression

The four evolving traits—energy‐acquisition rate a, PMRN intercept u, reproductive allocation c, and spawning duration $t_{\mathrm{sg}}$—are individually inherited. Parents for each offspring are selected using a von Neumann rejection algorithm (von Neumann, 1951) based on their reproductive success (Equation 3.1 for males and Equation A7 for females). The inherited genetic trait values $x_{g}$ (where x denotes any one of the four evolving traits) are sampled from a normal probability density function N with a mean given by the mid‐parental value (defined as the arithmetic mean of the maternal and paternal genetic trait values, $x_{\mathrm{mp}}=\left(x_{f,g}+x_{m,g}\right)/2$) and a variance $\sigma_{g,x}^{2}$ derived from the population's current mean $\mu_{g,x}$ of $x_{g}$ and a constant coefficient of variation CV,

(A15)
$$x_{g}\sim N\left(x_{\mathrm{mp}},,,\sigma_{g,x}^{2}\right)=N\left(\frac{x_{f,g}+x_{m,g}}{2},{\left(\mu_{g,x}\mathrm{CV}\right)}^{2}\right).$$

These genetic trait values $x_{g}$ are expressed as phenotypic trait values x by sampling from a normal probability density function with a mean given by the genetic trait value and a variance $\sigma_{e,x}^{2}$ derived from $\sigma_{g,x}^{2}$ according to the narrow‐sense heritability $h^{2}$,

(A16)
$$\begin{array}{c} x∼N\left(x_{g},,,\sigma_{e,x}^{2}\right)=N\left(x_{g},,,\frac{1-h^{2}}{h^{2}}{\left(\mu_{g,x}\mathrm{CV}\right)}^{2}\right). \end{array}$$

We assume $h^{2}$ to be constant, at a level generally expected for various life‐history traits in fish (Roff, 1991).

## APPENDIX 2

### Empirical data

Length at age was estimated from market data and survey data covering the full age and size range of North Sea plaice, taking account of the underlying size‐stratified sampling. Female reproductive investment was estimated as the sum of the ripening ovary weights and migration costs estimated at 13% of the body weight (Mollet et al., 2010). Somatic and reproductive tissue weights were standardized by the conversion ratio κ of the energy densities of gonadic to somatic tissue (κ=1.75 for North Sea plaice; Dawson et al., 1980).

Probabilistic maturation reaction norms (PMRNs) $p_{\mathrm{mat}}\left(t,l\right)$ are defined as the probability that an individual is becoming mature conditional on its age and size (Heino et al., 2002; Dieckmann & Heino, 2007; Heino & Dieckmann, 2008). They were derived from the maturity ogives $o\left(t,l\right)$, denoting the probability that an individual is being mature at age t and length l, estimated from the aforementioned data according to

(B1)
$$\text{logit}\left(o\left(t,l\right)\right)=\beta_{0}+\beta_{1}t+\beta_{2}l+\beta_{3}\mathrm{tl}+\varepsilon ,$$

with $\text{logit}\left(o\right)=\ln\left(o/\left(1-o\right)\right)$. While PMRNs in empirical studies are often defined retrospectively, as the probability $p_{\mathrm{mat}}$ of maturing at age t as a function of the change in the probability of being mature from age t−1year to age t (Barot et al., 2004), it is defined here prospectively to facilitate implementing the dependence of growth on maturity,

(B2)
$$p_{\mathrm{mat}}\left(t,l\right)=\frac{o\left(t+1,\text{year},l+\mathrm{\Delta l}\right)-o\left(t,l\right)}{1-o\left(t,l\right)},$$

where Δl is the expected length increment from age t to age t+1year.

Using the PMRNs estimated by decade according to Equations B1 and B2, the PMRN slope s was obtained by averaging the slopes estimated by decade. The empirically estimated PMRN midpoints are defined at each age t by the length l at which the maturation probability $p_{\mathrm{mat}}\left(t,l\right)$ estimated according to Equations B1 and B2 equals 50%. The PMRN intercept u was obtained by fitting through a linear regression, again by decade, the theoretically assumed PMRN midpoints (Equation 2) to the empirically estimated PMRN midpoints for the maturation‐relevant ages (present population: ages 2–4 years; historic population: ages 4–6 years) and averaging the results by decade. The empirically estimated PMRN widths are defined at each age t by the difference in length l over which the maturation probability $p_{\mathrm{mat}}\left(t,l\right)$ estimated according to Equations B1 and B2 changes between $p_{\mathrm{env}}=75\%$ and $1-p_{\mathrm{env}}=25\%$ (defining the maturation envelope's upper and lower bounds, respectively). The PMRN width d was obtained by averaging the PMRN widths estimated by decade. The scaling factor ϵ in Equation A5 was derived from the PMRN width d according to

(B3)
$$\epsilon =\frac{d}{2\;\text{logit}\left(p_{\mathrm{env}}\right)}.$$

## APPENDIX 3

### Sensitivity analysis

Our sensitivity analysis in Figure A2 reveals that the life histories generated by our model are most sensitive to changes in the exponent β of the allometric relationship between length and weight (Equation A3), the size‐specific maintenance rate b (Equations 1, A1 and A2), the exponent η of the allometric relationship between predation mortality and weight (Equation A10), the parameters υ and ω of the growth‐survival tradeoff (Equation A11), and the parameters $F_{\max}$ and λψ determining fishing mortality (Equation A14).

Of these parameters shared by both sexes, only η has opposite sex‐specific effects, due to male spawning mortality. For males, the life histories generated by our model are additionally sensitive to the proportionality constant θ between the reproductive‐investment rate and spawning duration (Equation 3.4) and the constant $\gamma_{50}$ describing the strength of diminishing returns in reproductive investment (Equation 3.2).

Generally, changes resulting in larger length at age also result in higher reproductive investment and later maturation, and vice versa (Figure A2).
